# Neuroprotective Effect of Ulinastatin on Spinal Cord Ischemia-Reperfusion Injury in Rabbits

**DOI:** 10.1155/2015/624819

**Published:** 2015-06-16

**Authors:** Bingbing Liu, Weihua Huang, Xiaoshan Xiao, Yao Xu, Songmei Ma, Zhengyuan Xia

**Affiliations:** ^1^Department of Anesthesiology, The 2nd People's Hospital of Guangdong Province, Guangdong Provincial Emergency Hospital, Guangzhou 510317, China; ^2^Department of Anesthesiology, Huizhou Municipal Central Hospital, Huizhou 516001, China; ^3^Department of Anesthesiology, The First People's Hospital of Shangqiu, Shangqiu 476000, China; ^4^Department of Anesthesiology, Affiliated Hospital of Guangdong Medical College, Zhanjiang 524003, China; ^5^Department of Anesthesiology, The University of Hong Kong, Hong Kong

## Abstract

Ulinastatin (UTI), a trypsin inhibitor, is isolated and purified from human urine and has been shown to exert protective effect on myocardial ischemia reperfusion injury in patients. The present study was aimed at investigating the effect of ulinastatin on neurologic functions after spinal cord ischemia reperfusion injury and the underlying mechanism. The spinal cord IR model was achieved by occluding the aorta just caudal to the left renal artery with a bulldog clamp. The drugs were administered immediately after the clamp was removed. The animals were terminated 48 hours after reperfusion. Neuronal function was evaluated with the Tarlov Scoring System. Spinal cord segments between L_2_ and L_5_ were harvested for pathological and biochemical analysis. Ulinastatin administration significantly improved postischemic neurologic function with concomitant reduction of apoptotic cell death. In addition, ulinastatin treatment increased SOD activity and decreased MDA content in the spinal cord tissue. Also, ulinastatin treatment suppressed the protein expressions of Bax and caspase-3 but enhanced Bcl-2 protein expression. These results suggest that ulinastatin significantly attenuates spinal cord ischemia-reperfusion injury and improves postischemic neuronal function and that this protection might be attributable to its antioxidant and antiapoptotic properties.

## 1. Introduction

Spinal cord injury is mainly divided into primary and secondary injuries according to pathophysiologic features. Primary injury mainly includes direct injury and ischemic injury, and it often occurs in a relatively short time after injury (generally after 4 hours of injury), with the irreversible nerve damage [1]. The secondary injury often takes place in the process of perfusion after spinal cord ischemia, causing spinal cord ischemia-reperfusion injury (SCIRI) which aggravates the neurofunctional damages of limbs. Spinal cord ischemia-reperfusion injury remains to be a devastating complication of thoracic aortic intervention, which may cause delayed paraplegia [2, 3]. The reasons for SCIRI were generally considered to be attributable to oxygen free radical-induced lipid peroxidation, leukocyte activation, inflammatory and neuronal apoptosis, and so on. Although technological advancements in surgery, such as hypothermic circulatory arrest, left heart bypass, intercostal artery reimplantation, and lumbar drains, have partly reduced complications of spinal cord injury, the incidence of paraplegia (immediate and delayed combined) after thoracic aortic intervention still ranges between 4% and 11% [4, 5]. Unfortunately, a reliable preventive method has not proven clinically efficacious in attenuating this injury until now. The mechanisms associated with delayed paraplegia are still not fully defined. Existing studies indicated that the apoptosis of motor neurons may cause the delayed paraplegia [6], and other studies showed that the cytokine interleukin- (IL-) 17 may play an important role in promoting spinal cord neuroinflammation after SCI via activation of STAT3 [7]. Therefore, it is necessary to formulate treatment tragedy for inhibiting the motor neurons apoptosis.

The urinary trypsin inhibitor, also called ulinastatin, is a protease inhibitor that is purified from human urine with a molecular weight of 67 kDa and it has been shown to have anti-inflammatory effect by suppressing the production of proinflammatory cytokines [8–10] and attenuated postoperative clinical outcome after myocardial ischemia-reperfusion in patients [11]. Ulinastatin significantly reduced pulmonary vascular permeability index (PVPI) and extravascular lung water index (ELWI) through inhibition of lipid peroxidation [12]. Experimental studies have shown organ protective effect of ulinastatin on ischemia-reperfusion injury of the lung, liver, heart, and kidney [13–16]. Ulinastatin also attenuates focal ischemia-reperfusion injury in rat brain, decreasing neutrophil infiltration in the ischemic hemisphere [17]. To the best of our knowledge, it is unknown whether ulinastatin has neuroprotective effects on spinal cord ischemia-reperfusion injury.

The aim of this study was to investigate the potential protective effects of ulinastatin on spinal cord ischemia-reperfusion injury and to explore its mechanism in relation to inhibiting neuron apoptosis in a rabbit model.

## 2. Materials and Methods

### 2.1. Animals

Adult male New Zealand White rabbits weighing 2.5–3.0 kg were provided by the Laboratory Animal Center of Southern Medical University (Guangzhou, China). Animals were housed at 22–25°C with a 12 h light/dark cycle before and after surgery. Standard animal chow and water were freely accessible. All experimental protocols were approved by the Institutional Animal Care and Use Committee of Southern Medical University and performed in accordance with the National Institutes of Health (NIH, USA) guidelines for the use of experimental animals. The rabbits were randomized and blindly assigned to three groups, with eight rabbits per group.

### 2.2. Experimental Grouping

The groups were as follows.

Sham control group (Sham, *n* = 8): rabbits underwent laminectomy and abdominal aorta was exposed but with no aortic occlusion clamp.

Ischemia-reperfusion (IR) group (IR, *n* = 8): rabbits underwent transient global spinal cord ischemia; the saline (0.9% NaCl, 5 mL/kg) was injected intravenously immediately after the occlusion clamp was removed.

Ulinastatin group (IR + UTI, *n* = 8): as for ischemia group, but ulinastatin at the dose of 50,000 U/kg (diluted with saline (0.9% NaCl) to 5 mL per kilogram) was injected intravenously immediately after the occlusion clamp was removed.

### 2.3. Experimental Protocols

Anesthesia was induced by intraperitoneal administration of 30 mg/kg ketamine. When necessary, additional dose of ketamine was administrated. A 22 G catheter was inserted into ear artery for measuring the arterial pressure. An ear vein catheter was placed for administration of additional medications and fluids. The anesthesia was maintained by intermittent intravenous injection of ketamine. The lactated Ringer's solution (10 mL/kg/h) was intravenously infused. The animals were intubated, placed supine on a heated operating table, and ventilated with 90% oxygen. Core body temperature was maintained at 36 ± 0.5°C. Animals were placed in the supine position for the surgery. After sterile preparation, a 10 cm midline incision was made, and the abdominal aorta was exposed through a transperitoneal approach. Heparin (130 U/kg) was administered intravenously 5 min before clamping for anticoagulation. Approximately 1 cm below the left renal artery, the aorta was clamped using a bulldog clamp. Ischemia-reperfusion injury was achieved via occlusion of the abdominal aorta for 40 min, and then the clamp was removed. After the surgical and ischemic interventions, the surgical wound was closed in layers with 3-0 silk sutures. The animals were given free access to water and food at room temperature.

At 48 hours after reperfusion, the animals were euthanized under deep anesthesia and transcardiac perfusion with 500 mL ice-cold saline was performed. The lumbar spinal cord (L_3–5_ segments) of all animals was removed. Spinal cord samples were carefully dissected and divided into two sections. One of the sections was placed in 4% paraformaldehyde for 24 hours. Following fixation, tissue sample was embedded in paraffin. The other part of tissue sample was flash frozen in liquid nitrogen and stored at −80°C until further analysis.

### 2.4. Functional Assessment

Neurologic function was scored at 4, 12, 24, and 48 hours after reperfusion by assessing hind-limb neurologic function using the Tarlov Scoring System. A score of 0 to 4 was assigned to each animal as follows: 0 = spastic paraplegia and no movement of the lower limbs, 1 = spastic paraplegia and slight movement of the lower limbs, 2 = good movement of the lower limbs but unable to stand, 3 = able to stand but unable to walk normally, and 4 = complete recovery and normal gait-hopping. Neurologic function evaluation was performed by a medical doctor who was blinded to the experimental groups.

### 2.5. Assay of SOD and MDA Activities

For biochemical analysis, spinal cord tissues were washed two times with cold saline solution and stored in −80°C until analysis. Tissue malondialdehyde (MDA) levels were determined as described [18]. Briefly, MDA was reacted with thiobarbituric acid by incubating for 1 hour at 95–100°C and fluorescence intensity was measured in the n-butanol phase with a fluorescence spectrophotometry (Hitachi, Mode l F-4010, Japan), by comparing with a standard solution of 1,1,3,3-tetramethoxypropane. The results were expressed in terms of nmol/g wet tissue. Total (Cu–Zn and Mn) superoxide dismutase (SOD) activity was measured according to reduction of nitro-blue tetrazolium by xanthine-xanthine oxidase system as described previously. Enzyme activity leading to 50% inhibition was regarded as one unit. The results were expressed as U/mg of protein. Protein concentrations were determined according to Lowry's method [19].

### 2.6. Determination of the Expression of Bcl-2, Bax, and Caspase-3 by Immunohistochemistry

The spinal cord tissue sections were deparaffinized in xylene and immersed in graded ethanol and distilled water. Immunohistochemical staining was performed using the avidin-biotin peroxidase complex (ABC) method according to the manufacturer's instructions (Dako, Carpinteria, CA, USA). The sections were incubated with a mouse monoclonal anti-Bax IgG2b antibody (1 : 500, sc-7480, Santa Cruz Biotechnology, CA, USA), a mouse monoclonal anti-Bcl-2 IgG1 antibody (1 : 20, sc-8392, Santa Cruz Biotechnology, CA, USA), and caspase-3 (1 : 500, Santa Cruz Biotechnology, Inc. Santa Cruz, CA, USA). The primary antibody was omitted as a negative control for the immunostaining. An image of each section was captured using a light microscope (Canon, Tokyo, Japan) at ×400 magnification and the integrated optical density (IOD) of the positively stained tissue in each image was determined using Image Pro Plus software, version 6.0 (Media Cybernetics, Silver Spring, MD, USA).

### 2.7. Determination of the Expression of Bcl-2, Bax, and Caspase-3 Protein by Western Blot

Frozen spinal cord samples were processed for protein assays using standard Western blotting analysis as described [20, 21]. In brief, the samples were homogenized in 5 v of buffer containing buffer containing sucrose 300 mM, HEPES 4 mM, EGTA 2 mM, phenylmethylsulphonyl fluoride (PMSF) 1 mM, and leupeptin 20 mM using a polytron homogenizer at the maximum speed in five 5 s bursts. The homogenates were incubated at 4°C for 30 min and then centrifuged at 10,000 ×g for 30 min at 4°C. The lysates were collected and the protein concentration was determined using the BCA Protein Assay kit. Equal amounts of proteins were separated by SDS-PAGE and transferred to polyvinylidene difluoride membranes (GE Healthcare Biosciences). The blots were initially blocked overnight with 5% milk in buffer containing Tris-HCl 20 mM (pH 7.4), NaCl 137 mM, 0.05% Tween-20, and then incubated for 1 h with anti-Bcl-2 antibody (1 : 1000), anti-Bax antibody (1 : 1000), anti-caspase-3 antibody (1 : 1000), all from Santa Cruz Biotechnology, CA, USA. After washing, the blots were incubated for 1 h at room temperature with a peroxidase-linked, goat anti-mouse secondary antibody (1 : 1000 dilution). The internal control was monoclonal anti-actin antibody (Sigma, St. Louis, USA) diluted 1 : 250. The bound antibody was then visualized using an enhanced chemiluminescence (ELC) kit (Amersham). Bcl-2, Bax, and caspase-3 were quantified densitometrically using suitable autoradiographs sucrose 300 mM, HEPES 4 mM, EGTA. Immunoblot results were quantified with Gel-Pro Analyzer 4.0 software (Media Cybernetics, Silver Spring, MD, USA).

### 2.8. Terminal Deoxynucleotidyl Transferase-Mediated dUTP-Biotin Nick End Labeling (TUNEL) Assay

Fixed spinal cord slices were embedded in paraffin, and 4 mm thick sections were deparaffinized by washing in 100% xylene and a descending ethanol series (from 100% ethanol two times to 80% ethanol once and 60% ethanol once). The sections were stained with haematoxylin and processed as described [22]. DNA fragments were determined using an ApopTag* in situ* apoptosis detection kit (ApopTag, Oncor, USA). The DNA nick was labelled according to the manufacturer's instructions. Following TUNEL, the sections were counterstained with haematoxylin. Neurons in which the nucleus was obviously labeled with diaminobenzidine were defined as TUNEL-positive. The apoptotic index (AI) was used to quantify the number of TUNEL positive cells. Five nonadjacent fields in each section were randomly chosen to count the total number of neurons and positive cells. The AI was calculated as follows: AI = (number of apoptotic cells/total number counted) × 100%.

### 2.9. Statistical Analysis

All data are expressed as the mean ± standard deviation (SD). Parametric statistics analyses were performed by ANOVA followed by Dennett's test for multiple comparisons or by Student's *t*-test. Nonparametric statistics analyses were performed by Kruskal-Wallis test followed by the Mann-Whitney *U* test with Bonferroni correction. *P* values less than 0.05 were considered significant. Statistical analysis was performed using the Statistical Package for the Social Sciences version 13.0 program (SPSS, Chicago, IL, USA).

## 3. Results

### 3.1. Neurologic Function

**Figure 1:**
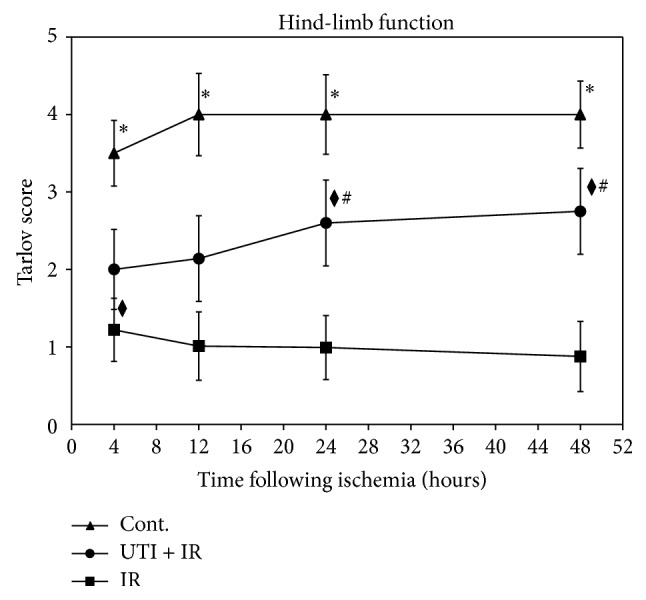
Hind-limb function indicated a progressive decline in function of ischemia-reperfusion IR controls. The scores of Sham group at the same time point were higher than UTI + IR and IR groups (^*^ *P* < 0.05). The scores of hind-limb function in IR group declined from 4 h to 48 h, but rabbits treated with UTI (UTI + IR group) did not decline; their function stabilized and was significantly greater (^#^ *P* < 0.01) than that in the IR controls at 24 h and 48 h after reperfusion. In UTI + IR group, function score was higher at 24 h and 48 h time points than at 4 h and 12 h time points (^⧫^ *P* < 0.05), but the score of IR group was higher at 4 h time point than at the other time points (^⧫^ *P* < 0.05). Values are the means ± SD, *n* = 8 per group.

Hind-limb function was recorded using the Tarlov Scoring System (Figure 1). In the Sham group, the scores from 4 to 48 h after reperfusion did not significantly change (*P* > 0.05), but in the UTI + IR group the scores were higher at 24 h and 48 h time points than at 4 h and 12 h time points (*P* < 0.05). The score in the IR group was higher 4 h after reperfusion than 12 to 48 h (*P* < 0.05). At 24 h and 48 h time points, the hind-limb function of UTI + IR group animals was improved compared to that of IR group animals (*P* < 0.01), but it did not reach the level of Sham group (*P* < 0.05).

### 3.2. Effect of Ulinastatin on MDA, SOD Levels

**Figure 2:**
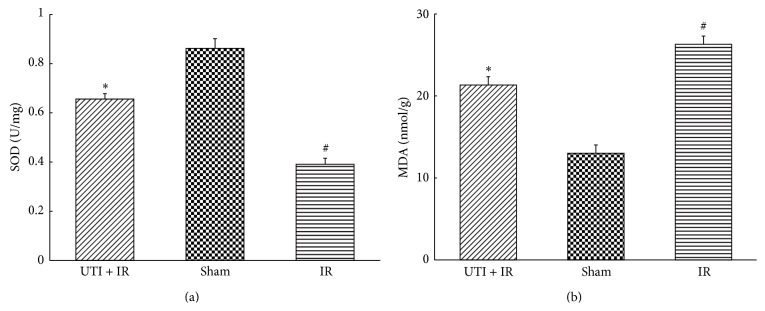
(a) SOD activities and MDA levels in rabbit spinal cord.  ^*^Compared with IR group (*P* < 0.01).  ^#^Compared with Sham group (*P* < 0.01). Values are the means ± SD, *n* = 8 per group.

To determine the local oxidative/antioxidative levels, we detected the MDA content and SOD activities in spinal cord tissue. Spinal cord IR was associated with significant elevation in MDA production with concomitant reduction in SOD activity. Following ischemia-reperfusion injury, the SOD activity in the UTI + IR group was significantly higher than that in the IR group, but MDA content in spinal cord tissue was decreased significantly compared with IR group (Figure 2).

### 3.3. Immunohistochemistry of Bcl-2, Bax, and Caspase-3 Proteins

**Figure 3:**
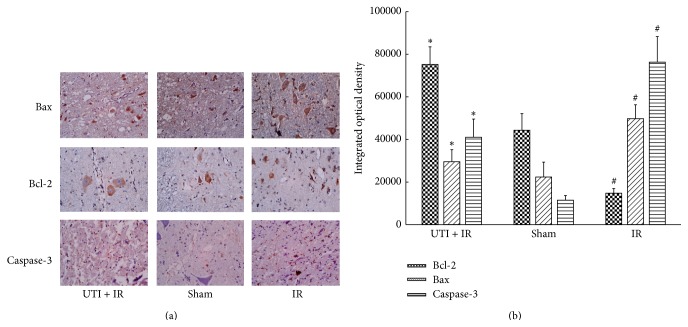
The expression of Bax, Bcl-2, and caspase-3 detected by immunohistochemistry. IR injury promoted Bax and caspase-3 expression but suppressed Bcl-2 expression. However, ulinastatin suppressed Bax and caspase-3 expression and promoted Bcl-2 expression.  ^*^Compared with IR group (*P* < 0.01).  ^#^Compared with Sham group (*P* < 0.01). Values are the means ± SD, *n* = 8 per group.

As shown in Figure 3, spinal cord ischemia-reperfusion resulted in significant increase of Bax and caspase-3 protein expression and decrease of Bcl-2 protein expression as compared with the Sham group. The number of the positive cells containing Bcl-2 protein expression (brown stain) increased in the UTI + IR group compared to the IR control group. The number of positive cells containing Bax and caspase-3 protein expression (brown stain) decreased in the UTI + IR group compared with the IR group.

### 3.4. Western Blot Analysis of Bcl-2, Bax, and Caspase-3 Proteins

**Figure 4:**
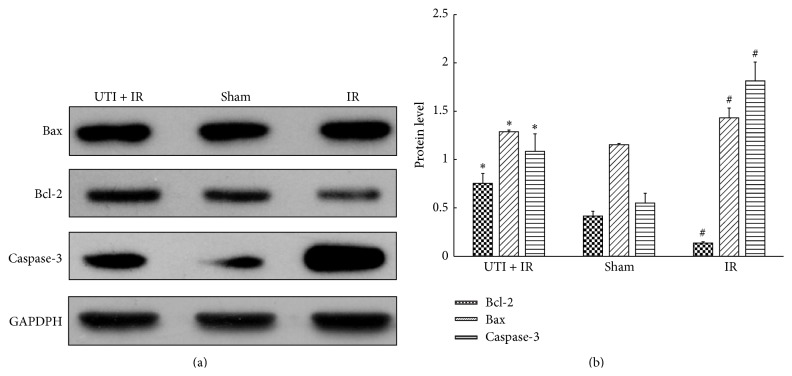
Expression level of apoptosis relevant proteins. (a) Western blot identified the expression level of apoptosis relevant proteins: Bax, Bcl-2, and caspase-3 in the spinal cord after 48 hours of reperfusion. (b) Compared with the Sham group, the IR group showed a significant increase of Bax, caspase-3 but decrease of Bcl-2. Compared with the IR group, the UTI + IR group demonstrated a significant decreased of Bax and caspase-3 but increase of Bcl-2.  ^*^Compared with IR group (*P* < 0.01).  ^#^Compared with Sham group (*P* < 0.01). Values are the means ± SD, *n* = 8 per group.

The expression of Bax protein was significantly upregulated in the IR control group as compared to the Sham group. However, this upregulation of the expression of Bax protein was significantly inhibited in the IR + UTI group. With respect to Bcl-2, the expression level in the IR + UTI group significantly increased as compared with the Sham group and the IR group, respectively. The expression of caspase-3 decreased in the IR + UTI group, but increased in the IR control group (Figure 4).

### 3.5. Apoptotic Cell Death Assessed by TUNEL Staining

**Figure 5:**
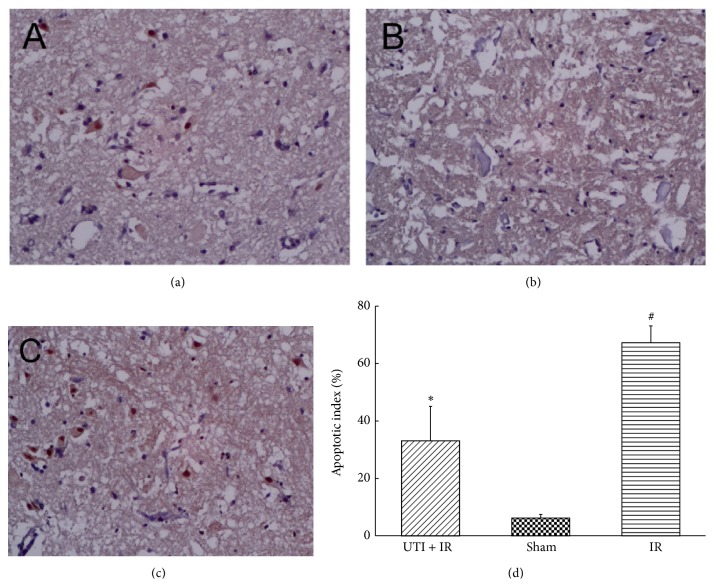
TUNEL staining and the apoptotic index of spinal cord neurons. (a) In the UTI + IR group, the number of apoptotic cells was far less than that in the IR group, which suggested that UTI decreased cell apoptosis caused by IR. (b) In the Sham group, a very few apoptotic cells were found. (c) In the IR group, the apoptotic cells increased significantly.  ^*^Compared with IR group (*P* < 0.01).  ^#^Compared with Sham group (*P* < 0.01). Values are the means ± SD, *n* = 8 per group.

TUNEL-positive cells were minimally detectable in the Sham group, whereas the IR group showed a significant number of TUNEL-positive cells. Ulinastatin intervention significantly decreased the number of TUNEL-positive cells compared to the IR group (*P* < 0.05, Figure 5).

## 4. Discussion

The aim of this study was to investigate the potential protective effects of ulinastatin on spinal cord ischemia-reperfusion injury and to explore the underlying mechanisms. The present study showed that ulinastatin improved the neurological outcome and reduced the postischemic apoptotic cell death in neurons by way of decreasing the levels of  MDA, increasing SOD activities, suppressing the upregulation of the proapoptotic protein expression and the downregulation of antiapoptotic protein expression.

Spinal cord ischemia and reperfusion injury is a common postoperative complication following surgeries implicating the descending and thoracoabdominal aorta, which may lead to catastrophic consequences, such as paraplegia. Spinal cord ischemia due to hypoperfusion during aortic cross clamping is largely responsible for spinal cord injury. This injury is followed by a secondary injury caused by blood reperfusion [23]. The lack of the nutrients and oxygen activates devastating biochemical cascades, which causes spinal cord ischemia and reperfusion injury, namely, primary injury [24]. The primary injury followed by blood reperfusion may cause additional spinal cord damage, which impairs neuron function and generates the secondary damage. Secondary damage to the spinal cord is primarily responsible for many negative effects of the spinal cord injury (SCI) and most researches however have focused on understanding the pathophysiology of the secondary damage and reducing the amount of delayed cell loss following SCI [25, 26]. It has been reported that production of reactive oxygen species (ROS) is a well characterized pathological process during the reperfusion [27]. The event triggers accumulation of intracellular calcium levels, mitochondrial dysfunction [23]. ROS causes lipid peroxidation as well as oxidative and nitrative damage to proteins and nucleic acids [28, 29]. The central nervous system is primarily composed of lipids, which makes it easily damaged by free-radical-induced lipid peroxidation [30]. Lipid peroxidation is believed to be one of the primary pathophysiological mechanisms, which is also implicated in secondary damage [31]. Under the normal conditions, there is a balance between the production of ROS and the defense system, the antioxidants. Once the production of ROS is beyond the capacity of those antioxidant enzymes (such as SOD) as happened during reperfusion, oxidative damage occurred.

Ulinastatin, a protease inhibitor, was a glycoprotein, which was obtained from human urine with a molecular weight of 67 kDa [32], and it has been showed to reduce lipid peroxidation in various models of ischemia-reperfusion or sepsis [33]. In the present study, ulinastatin treatment attenuated lipid peroxidation by decreasing MDA content and elevating SOD activity in the spinal cord tissue, suggesting that ulinastatin can attenuate oxidative stress of the spinal cord tissue. Ulinastatin produced dose-dependent attenuation of the systemic inflammatory response of rats following lung I/R injury [34], and the doses of ulinastatin had been used from 5000 U/kg to 300000 U/kg [17, 34]. Therefore, we chose 50000 U/kg as the treatment dose, and our data further confirm that ulinastatin inhibits free radicals induced oxidative stress by accelerating its scavenging and thus plays a protective role after spinal cord ischemia reperfusion.

Evaluation of neurologic function is a current means for accessing the degree of the injury and the outcome of a medication treatment. The Sham group did not have nerve function impairment as assessed by using the Basso, Beattie, and Bresnahan (BBB) hind-limb locomotor rating scale test. However, in the SCI group the animals became unconscious at 1 hour time point and their BBB scores increased from 1.41 at 24 h to 4.51 at 72 h [7]. Similarly, in the present study the Sham group hind-limb function was also normal at the different time points, but the function in the IR group was impaired from 12 h to 48 h after reperfusion.

Apoptosis is the process of programmed cell death, which is indispensable for the normal development and homeostasis of all multicellular organisms [35]. It was believed that neuron apoptosis played a pivotal role in the second injury after spinal cord ischemia reperfusion. Previous research has demonstrated that a line of genes are involved in the development of apoptosis, such as the Bcl-2 gene family. The Bcl-2 gene family consists of 12 different gene products with pro- and antiapoptotic mechanisms. These pro- and antiapoptotic proteins are different with regard to structure and tissue distribution and exert different functional effects. Bcl-2 and Bax are essentially involved in the regulation of cell apoptosis [36]. The antiapoptotic protein Bcl-2 is localized mainly in the outer mitochondrial membrane. In contrast, the proapoptotic proteins Bax reside mainly in the cytoplasm and are activated by various apoptotic stimuli. Bax translocates to the mitochondria, where it forms a complex with Bcl-2. An increased ratio of Bax/Bcl-2 leads to the formation of pores in the mitochondria, release of cytochrome c, and activation of the apoptotic pathway. Bcl-2 can prevent the release of cytochrome c, along with dATP and Apaf-1, and thus protect from apoptosis [37]. Chen et al. [38] reported that ulinastatin reduced the renal dysfunction and injury associated with ischemia-reperfusion of the kidney by upregulating of Bcl-2 expression. In our study, the TUNEL staining indicated that ulinastatin significantly reduced neuronal apoptosis in spinal cord after spinal cord ischemia reperfusion. Ulinastatin significantly upregulated Bcl-2 expression while suppressing the overexpression of Bax, suggesting that ulinastatin has an antiapoptosis property that plays a protective role attenuating postischemic spinal cord injury.

Caspases are a family of inactive proenzymes that play a crucial role in cell apoptosis, which is the scheduled death of cells. Caspase-3 is a protein that regulates apoptosis by inducing the cleavage of the key cellular proteins and alters cell integrity [39]. The role of caspase-3 in apoptosis is to activate the stages of cellular death in a nontraumatic manner. The intrinsic apoptosis is triggered by a wide range of stimuli that leads to release of cytochrome c from the mitochondria. This results in the formation of the apoptosome. The apoptosome then activates initiator caspase, typically caspase-9, which leads to the activation of the executioner caspase-3, and apoptosis finally occurs. Caspase-3 as the executioner is the common pathway which must be passed through in the apoptosis cascade, and it is the key one in mammalian cell apoptosis [40]. Caspase-3 is vital to this process because it allows the process to progress in sequence, which prevents undue damage to the remaining cells. In the present study, we found that the expression of caspase-3 was elevated in the IR group, but ulinastatin could decrease it significantly; thus the protective effect of ulinastatin may be in part due to inhibition of caspase-3 protein expression.

In summary, our results demonstrate that ulinastatin improves postischemic neurological function. To be best of our knowledge, the current study is the first to investigate the effects and mechanisms of ulinastatin in attenuating spinal cord ischemia-reperfusion injury. We have shown that ulinastatin increases SOD activity and decreases MDA content. Finally, we have revealed that ulinastatin can inhibit the neuronal apoptosis by regulating the expression of apoptosis proteins including Bcl-2, Bax, and caspase-3. Therefore, we propose that ulinastatin has a protective role and improves neurological outcome due to its beneficial effects including antioxidant and antiapoptosis effects. Findings obtained from the current study may have significant clinical implications given that ulinastatin is in use clinically.

## References

[1] B-Rao C., Stewart J. (1996). Inverse analysis of empirical matrices of idiotypic network interactions. *Bulletin of Mathematical Biology*.

[2] Smith P. D., Puskas F., Meng X. (2012). The evolution of chemokine release supports a bimodal mechanism of spinal cord ischemia and reperfusion injury. *Circulation*.

[3] Kakinohana M., Kida K., Minamishima S. (2011). Delayed paraplegia after spinal cord ischemic injury requires caspase-3 activation in mice. *Stroke*.

[4] Lang-Lazdunski L., Matsushita K., Hirt L., Waeber C., Vonsattel J.-P. G., Moskowitz M. A. (2000). Spinal cord ischemia: development of a model in the mouse. *Stroke*.

[5] Conrad M. F., Ye J. Y., Chung T. K., Davison J. K., Cambria R. P. (2008). Spinal cord complications after thoracic aortic surgery: long-term survival and functional status varies with deficit severity. *Journal of Vascular Surgery*.

[6] Hayashi T., Sakurai M., Abe K., Sadahiro M., Tabayashi K., Itoyama Y. (1998). Apoptosis of motor neurons with induction of caspases in the spinal cord after ischemia. *Stroke*.

[7] Zong S., Zeng G., Fang Y. (2014). The role of IL-17 promotes spinal cord neuroinflammation via activation of the transcription factor STAT3 after spinal cord injury in the rat. *Mediators of Inflammation*.

[8] Oda J., Yamashita K., Inoue T. (2006). Resuscitation fluid volume and abdominal compartment syndrome in patients with major burns. *Burns*.

[9] Endo S., Inada K., Taki K., Hoshi S., Yoshida M. (1990). Inhibitory effects of ulinastatin on the production of cytokines: implications for the prevention of septicemic shock. *Clinical Therapeutics*.

[10] Cao Y.-Z., Tu Y.-Y., Chen X., Wang B.-L., Zhong Y.-X., Liu M.-H. (2012). Protective effect of Ulinastatin against murine models of sepsis: inhibition of TNF-*α* and IL-6 and augmentation of IL-10 and IL-13. *Experimental and Toxicologic Pathology*.

[11] He Q.-L., Zhong F., Ye F. (2014). Does intraoperative ulinastatin improve postoperative clinical outcomes in patients undergoing cardiac surgery: a meta-analysis of randomized controlled trials. *BioMed Research International*.

[12] Luo H.-M., Du M.-H., Lin Z.-L. (2013). Ulinastatin suppresses burn-induced lipid peroxidation and reduces fluid requirements in a swine model. *Oxidative Medicine and Cellular Longevity*.

[13] Cao Z.-L., Okazaki Y., Naito K., Ueno T., Natsuaki M., Itoh T. (2000). Ulinastatin attenuates reperfusion injury in the isolated blood-perfused rabbit heart. *Annals of Thoracic Surgery*.

[14] Binns O. A. R., DeLima N. F., Buchanan S. A. (1996). Neutrophil endopeptidase inhibitor improves pulmonary function during reperfusion after eighteen-hour preservation. *The Journal of Thoracic and Cardiovascular Surgery*.

[15] Kudo Y., Egashira T., Yamanaka Y. (1992). Protective effect of ulinastatin against liver injury caused by ischemia-reperfusion in rats. *Japanese Journal of Pharmacology*.

[16] Nakahama H., Obata K., Sugita M. (1996). Ulinastatin ameliorates acute ischemic renal injury in rats. *Renal Failure*.

[17] Yano T., Anraku S., Nakayama R., Ushijima K. (2003). Neuroprotective effect of urinary trypsin inhibitor against focal cerebral ischemia-reperfusion injury in rats. *Anesthesiology*.

[18] Yao W., Luo G., Zhu G. (2014). Propofol activation of the Nrf2 pathway is associated with amelioration of acute lung injury in a rat liver transplantation model. *Oxidative Medicine and Cellular Longevity*.

[19] Lowry O. H., Rosebrough N. J., Farr A. L., Randall R. J. (1951). Protein measurement with the Folin phenol reagent. *The Journal of Biological Chemistry*.

[20] Luo T., Xia Z., Ansley D. M. (2005). Propofol dose-dependently reduces tumor necrosis factor-*α*-induced human umbilical vein endothelial cell apoptosis: effects on Bcl-2 and bax expression and nitric oxide generation. *Anesthesia and Analgesia*.

[21] Liu M., Zhou B., Xia Z.-Y. (2013). Hyperglycemia-induced inhibition of DJ-1 expression compromised the effectiveness of ischemic postconditioning cardioprotection in rats. *Oxidative Medicine and Cellular Longevity*.

[22] Li H., Liu Z., Wang J. (2013). Susceptibility to myocardial ischemia reperfusion injury at early stage of type 1 diabetes in rats. *Cardiovascular Diabetology*.

[23] Kouchoukos N. T., Scharff J. R., Castner C. F. (2014). Repair of primary or complicated aortic coarctation in the adult with cardiopulmonary bypass and hypothermic circulatory arrest. *The Journal of Thoracic and Cardiovascular Surgery*.

[24] Kertmen H., Gürer B., Yilmaz E. R. (2013). The protective effect of low-dose methotrexate on ischemia-reperfusion injury of the rabbit spinal cord. *European Journal of Pharmacology*.

[25] Hall E. D., Springer J. E. (2004). Neuroprotection and acute spinal cord injury: a reappraisal. *NeuroRx*.

[26] Oyinbo C. A. (2011). Secondary injury mechanisms in traumatic spinal cord injury: a nugget of this multiply cascade. *Acta Neurobiologiae Experimentalis*.

[27] Azbill R. D., Mu X., Bruce-Keller A. J., Mattson M. P., Springer J. E. (1997). Impaired mitochondrial function, oxidative stress and altered antioxidant enzyme activities following traumatic spinal cord injury. *Brain Research*.

[28] Fan Q., Yang X.-C., Liu Y. (2011). Postconditioning attenuates myocardial injury by reducing nitro-oxidative stress in vivo in rats and in humans. *Clinical Science*.

[29] Lei S., Li H., Xu J. (2013). Hyperglycemia-induced protein kinase C *β*2 activation induces diastolic cardiac dysfunction in diabetic rats by impairing caveolin-3 expression and Akt/eNOS signaling. *Diabetes*.

[30] Yilmaz E. R., Kertmen H., Dolgun H. (2012). Effects of darbepoetin-alpha in spinal cord ischemia-reperfusion injury in the rabbit. *Acta Neurochirurgica*.

[31] Diaz-Ruiz A., Rios C., Duarte I. (2000). Lipid peroxidation inhibition in spinal cord injury: cyclosporin-A vs methylprednisolone. *NeuroReport*.

[32] Yamauchi Y., Izumi Y., Inoue M. (2011). Safety of postoperative administration of human urinary trypsin inhibitor in lung cancer patients with idiopathic pulmonary fibrosis. *PLoS ONE*.

[33] Koga Y., Fujita M., Tsuruta R. (2010). Urinary trypsin inhibitor suppresses excessive superoxide anion radical generation in blood, oxidative stress, early inflammation, and endothelial injury in forebrain ischemia/reperfusion rats. *Neurological Research*.

[34] Xu L., Ren B., Li M., Jiang F., Zhanng Z., Hu J. (2008). Ulinastatin suppresses systemic inflammatory response following lung ischemia-reperfusion injury in rats. *Transplantation Proceedings*.

[35] Kannan K., Jain S. K. (2000). Oxidative stress and apoptosis. *Pathophysiology*.

[36] Antonsson B., Martinou J. C. (2000). The Bcl-2 protein family. *Experimental Cell Research*.

[37] Green D. R., Reed J. C. (1998). Mitochondria and apoptosis. *Science*.

[38] Chen C.-C., Liu Z.-M., Wang H.-H., He W., Wang Y., Wu W.-D. (2004). Effects of ulinastatin on renal ischemia-reperfusion injury in rats. *Acta Pharmacologica Sinica*.

[39] Wang Y., Liu X., Zhang D., Chen J., Liu S., Berk M. (2013). The effects of apoptosis vulnerability markers on the myocardium in depression after myocardial infarction. *BMC Medicine*.

[40] Cohen G. M. (1997). Caspase: the executioners of apoptosis. *Biochemical Journal*.

